# Optimization of genomic selection training populations with a genetic algorithm

**DOI:** 10.1186/s12711-015-0116-6

**Published:** 2015-05-06

**Authors:** Deniz Akdemir, Julio I Sanchez, Jean-Luc Jannink

**Affiliations:** Department of Plant Breeding & Genetics, Cornell University, Ithaca, NY USA; Robert W. Holley Center for Agriculture and Health, USDA-ARS, Ithaca, NY USA

## Abstract

In this article, we imagine a breeding scenario with a population of individuals that have been genotyped but not phenotyped. We derived a computationally efficient statistic that uses this genetic information to measure the reliability of genomic estimated breeding values (GEBV) for a given set of individuals (test set) based on a training set of individuals. We used this reliability measure with a genetic algorithm scheme to find an optimized training set from a larger set of candidate individuals. This subset was phenotyped to create the training set that was used in a genomic selection model to estimate GEBV in the test set. Our results show that, compared to a random sample of the same size, the use of a set of individuals selected by our method improved accuracies. We implemented the proposed training selection methodology on four sets of data on Arabidopsis, wheat, rice and maize. This dynamic model building process that takes genotypes of the individuals in the test sample into account while selecting the training individuals improves the performance of genomic selection models.

**Electronic supplementary material:**

The online version of this article (doi:10.1186/s12711-015-0116-6) contains supplementary material, which is available to authorized users.

## Introduction

Genomic selection (GS) in animal or plant breeding is based on genomic estimated breeding values (GEBV). Prediction of GEBV involves a whole-genome regression model in which the known phenotypes are regressed on the markers. For breeding programs with limited phenotyping resources, the genotypic information can be used to select a ‘good’ training set of individuals to be phenotyped. Once the phenotypes are measured, a regression model can be trained to predict GEBV of individuals that have not been phenotyped. Since phenotyping is a time-consuming and costly process, selecting a ‘good’ training population is key to the success of GS.

In this article, we concentrate our efforts on the design of a training population to maximize the accuracy of the GS models. We have imagined a scenario in which two sets of individuals with genotypic information are available. The first set includes candidate individuals from which individuals for the training set are selected for phenotyping. The prediction model will be trained on this set. The second set is a test set on which the prediction model is validated or within which selection is applied to move breeding forward to the next cycle. We assume that genotyping information of genome-wide markers is available for all individuals. This scenario is very similar to that of breeding programs that use GS and calculate GEBV from a limited number of phenotypic observations. This scenario is especially useful when phenotyping is expensive and genotypic information is relatively affordable. In this work, we show that a model building process which takes genotypes of the individuals in the test sample into account while selecting the training individuals improves the performance of prediction models built on a random sample of the same size.

Note that our method of selection does not require any phenotypic information. It only requires genotypic information about the individuals in a candidate set and a test set (genome-wide markers or the relationship information given by a pedigree). Based on genotyping information, our method selects an optimized training population, which will be phenotyped after selection. In our study, the phenotypes are needed only to validate the benefits of the proposed method and to compare it to the random sample (correlation coefficients between GEBV and the observed phenotypes are calculated).

Various regression models have been successfully used to predict the breeding values in plants and animals [1,2]. In both simulation studies and empirical studies of dairy cattle, mice and in bi-parental populations of maize, barley and Arabidopsis, marker-based GEBV are quite accurate. However, as training and testing populations diverge, the accuracies of GEBV decreases [3,4]. Because breeding populations tend to change over time, accuracies of GEBV obtained from the training population decrease over time. Similarly, in the presence of strong population structure, GEBV obtained by using sub-populations are usually not accurate for individuals in other sub-populations.

In breeding, the design of training populations is an issue that has captured some attention [5,6]. The reliability measure of VanRaden [7] is expressed as:

(1) $$ \mathbf{K}_{21}(\mathbf{K}_{11}+\delta \mathbf{I})^{-1}\mathbf{K}^{\prime}_{21},  $$

where **K**_21_ is the matrix of genomic relationships between individuals in the test set with each of the individuals in the training set, **K**_11_ measures the genomic relationships in the training set and parameter *δ* is related to the heritability (*h*^2^) of the trait as follows *δ*=(1−*h*^2^)/*h*^2^. This reliability measure is related to Henderson’s prediction error variance (PEV) [8] and the more recent coefficient of determination (CD) of Laloë et al. [9]. These measures were used in [5] to investigate the issue on training population design.

Computational cost of calculating the reliability measure in Equation (1) and the related PEV and CD increase with sample size. Finding an optimal training population involves evaluating these measures many times which is not computationally feasible for large sample sizes [10]. Therefore, it is important to be able to estimate reliability efficiently. In the next section, we derive a computationally efficient approximation to the PEV based on the principal components of the genotypes and use this measure for training population design. Other efficient methods for the calculation of these statistics have been discussed in the literature [7,10,11].

Another major originality of our method compared to the optimization schemes recommended in [5,6] is that we calculate the PEV for the individuals in the test set instead of the candidate set, i.e., we use domain information about the test data while building the estimation model by choosing individuals for the training set such that they minimize the PEV in the test set. The methods developed here can be used for dynamic model building, in other words, for choosing different training sets to be phenotyped, and hence fitting different estimation models, as a function of the test set.

## Methods

Traditionally, the breeder is interested in the additive genetic or breeding value as opposed to the total genetic value. Therefore, a linear model is assumed between markers and phenotypes. This is expressed as:

$$y=\beta_{0}+\boldsymbol{m}^{\prime}\boldsymbol{\beta}+e, $$

where *y* stands for the phenotype, *β*_0_ is the mean parameter, ***m*** is the *m*−vector of marker genotypes, ***β*** is the *m*−vector of marker effects and *e* is the residual term which is assumed to follow a normal distribution with a 0 mean and variance ${\sigma _{e}^{2}}.$

In order to estimate the parameters of this model, we acquire observations on *n*_*Train*_ individuals from the larger candidate set. The model is used to generate predictions on a fixed set of *n*_*Test*_ individuals.

Let **M** be the centered and scaled matrix of marker genotypes partitioned as:

$$\mathbf{M}= \left[ \begin{array}{c} \frac{\mathbf{M}_{Candidate}} {\mathbf{M}_{Test}} \end{array} \right], $$

where **M**_*Candidate*_ is the *n*×*m* matrix of marker genotypes for the individuals in the candidate set and **M**_*Test*_ is the matrix of marker genotypes for the individuals in the test set. Our aim is to identify *n*_*Train*_ training set individuals from the candidate set (and therefore a matrix **M**_*Train*_) for which the average prediction variance for the individuals in the test set is minimized. Given that we have determined **M**_*Train*_ and their phenotypes ***y***_*Train*_ are available, we can write:

$$\boldsymbol{y}_{Train}=(\boldsymbol{1}, \mathbf{M}_{Train})(\beta_{0}, \boldsymbol{\beta}^{\prime})^{\prime}+\boldsymbol{e}. $$

Under the assumptions of this model, the uniformly minimum variance estimators for the phenotypes in the test data are expressed as:

(2) $$ \begin{aligned} &\widehat{\boldsymbol{y}}_{Test}\\ & \quad\,\,=(\boldsymbol{1}, \mathbf{M}_{Test})((\boldsymbol{1},\mathbf{M}_{Train})^{\prime}(\boldsymbol{1},\mathbf{M}_{Train}))^{-} \\ &\qquad (\boldsymbol{1},\mathbf{M}_{Train})^{\prime}\boldsymbol{y}_{Train}, \end{aligned}  $$

where the ^−^ denotes the pseudo-inverse of a matrix. Ignoring the constant term, $ {\sigma _{e}^{2}},$ the covariance matrix (PEV) for $ \widehat {\boldsymbol {y}}_{\textit {Test}}$ is

$$\begin{aligned} &PEV(\mathbf{M}_{Test})\\ & \qquad=(\boldsymbol{1}, \mathbf{M}_{Test})((\boldsymbol{1},\mathbf{M}_{Train})'(\boldsymbol{1},\mathbf{M}_{Train}))^{-} \\ & \qquad(\boldsymbol{1},\mathbf{M}_{Test})^{\prime}. \end{aligned} $$

With the emergence of modern genotyping technologies, the number of markers can vastly exceed the number of individuals. To overcome the problems due to large *m* with small *n* regressions, several methods such as variable selection, shrinkage of estimates, or a combination of both have been proposed [12,13]. These methods trade the decreasing variance to increasing bias due to shrinkage of individual marker effects to obtain a better overall prediction performance compared to the ordinary least squares solution given in Equation (2). Ridge regression [14] is a commonly used shrinkage method in GEBV prediction [15,16] and the PEV for the ridge regression is given by:

(3) $$ \begin{aligned} &PEV^{Ridge}(\mathbf{M}_{Test})=(\boldsymbol{1}, \mathbf{M}_{Test})\\ & \qquad((\boldsymbol{1},\mathbf{M}_{Train})^{\prime}(\boldsymbol{1},\mathbf{M}_{Train})+\lambda \mathbf{I})^{-1}(\boldsymbol{1},\mathbf{M}_{Test})^{\prime}, \end{aligned}  $$

for a choice of *λ*>0. In order to obtain minimum variance for our predictions in the test data set, we considered minimizing the scalar measure *tr*(*PEV*^*Ridge*^(*M*_*Test*_)) with respect to *M*_*Train*_ when selecting individuals for the training set.

We note that the *PEV*^*Ridge*^(**M**_*Test*_) is related to the reliability measure in Equation (1). First, note that:

$$\begin{aligned} &(\mathbf{M}^{\prime}_{Train}\mathbf{M}_{Train}+\lambda \mathbf{I})^{-1}\\ & \quad=\frac{1}{\lambda}\left(\mathbf{I}-\mathbf{M}^{\prime}_{Train}\left(\mathbf{M}_{Train}\mathbf{M}^{\prime}_{Train}+\lambda \mathbf{I}\right)\right.^{-1}\mathbf{M}_{Train}. \end{aligned} $$

Letting $ \delta =m\lambda, \mathbf {K}_{21}= \mathbf {M}_{\textit {Test}}\mathbf {M}^{\prime }_{\textit {Train}}/m, \mathbf {K}_{11}= \mathbf {M}_{\textit {Train}}\mathbf {M}^{\prime }_{\textit {Train}}/m$ and $\mathbf {K}_{22}= \mathbf {M}_{\textit {Test}}\mathbf {M}^{\prime }_{\textit {Test}}/m$ and using the Woodbury matrix identity [17]:

$$(\mathbf{A}+\mathbf{C}\mathbf{B}\mathbf{C}^{\prime})=\mathbf{A}^{-1}-\mathbf{A}^{-1}\mathbf{C}(\mathbf{A}^{-1}+\mathbf{C}^{\prime}\mathbf{A}^{-1}\mathbf{C})\mathbf{C}^{\prime}\mathbf{A}^{-1}, $$

at the third step below, we have

$$\begin{aligned} & PEV^{Ridge}(\mathbf{M}_{Test})\\ &\quad\,\,\,=\mathbf{M}_{Test}(\mathbf{M}_{Train}\mathbf{M}_{Train}+\lambda \mathbf{I})^{-1} \mathbf{M}^{\prime}_{Test}\\ &\quad\,\,\,=\mathbf{M}_{Test}\left(\lambda\left(\frac{\mathbf{M}^{\prime}_{Train}\mathbf{M}_{Train}}{\lambda}+\mathbf{I}\right)\right)^{-1}\mathbf{M}^{\prime}_{test}\\ &\quad\,\,\,=\frac{1}{\lambda}\mathbf{M}_{Test}\\ &\quad\,\,\,\left(\mathbf{I}-\mathbf{M}^{\prime}_{Train}\left(\mathbf{M}_{Train}\mathbf{M}^{\prime}_{Train}+\lambda \mathbf{I}\right)^{-1}\mathbf{M}_{Train}\right)\\ &\quad \,\,\,\mathbf{M}^{\prime}_{Test}\\ &\quad\,\,\,= \frac{1}{\lambda}\left[\mathbf{M}_{Test}\mathbf{M}^{\prime}_{Test} \right] \\ &\quad\,\,\,-\frac{1}{\lambda}\left[\mathbf{M}_{Test}\mathbf{M}^{\prime}_{Train}\left(\mathbf{M}_{Train}\mathbf{M}^{\prime}_{Train} +\lambda \mathbf{I}\right)^{-1} \right.\\ &\left.{\vphantom{\left(\mathbf{M}_{Train}\mathbf{M}^{\prime}_{Train} +\lambda \mathbf{I}\right)^{-1}}}\quad\,\,\,\mathbf{M}_{Train}\mathbf{M}^{\prime}_{Test}\right] \\ &\quad\,\,\,\propto \mathbf{K}_{22} -\mathbf{K}_{21}(\mathbf{K}_{11}+m\lambda \mathbf{I})^{-1}\mathbf{K}^{\prime}_{21}. \end{aligned} $$

Therefore, maximizing average reliability is equivalent to minimizing the total *PEV*^*Ridge*^ in Equation (3).

Since we are dealing with a large number of markers and since any optimization scheme would involve many evaluations of this objective function, the formula for the *PEV*^*Ridge*^(**M**_*Test*_) is difficult to use in practice. A more suitable numerically efficient approximation to *PEV*^*Ridge*^(**M**_*Test*_) can be obtained by using the first few principal components (PC) of the marker matrix **M** instead of **M** itself. Let **P** be the matrix of first *k*≤*min*(*m*,*n*) PC partitioned as:

$$\mathbf{P}=\left[ \begin{array}{c} \frac{\mathbf{P}_{Candidate}} {\mathbf{P}_{Test}} \end{array} \right], $$

where **P**_*Candidate*_ is the matrix of PC for the individuals in the candidate set and **P**_*Test*_ is the matrix of PC’s for the individuals in the test set. Now, *PEV*^*Ridge*^(**M**_*Test*_) can be approximated by:

(4) $$ \begin{aligned} &PEV^{Ridge}(\mathbf{M}_{Test})\\ & \quad\,\,\,\approx (\boldsymbol{1}, \mathbf{P}_{Test})((\boldsymbol{1}, \mathbf{P}_{Train})^{\prime}(\boldsymbol{1}, \mathbf{P}_{Train})+\lambda \mathbf{I})^{-1}\\ & \quad\,\,\,\,(\boldsymbol{1}, \mathbf{P}_{Test})^{\prime}. \end{aligned}  $$

This approximation involves the inversion of a *k*+1 dimensional matrix and is computationally efficient compared to the measures in Equations (3) and (1), which involve the inversion of *m*+1 and *n*_*Train*_ dimensional matrices.

Since many candidate training sets need to be evaluated in the course of optimization, we preferred the computationally efficient approximation in Equation (4) over the exact *PEV*^*Ridge*^(**M**_*Test*_). The scalar measure obtained by taking the trace of Equation (4) was used to evaluate training populations subsequently.

Numerous algorithms have been proposed for optimal design. Most of these approaches combine heuristics with an exchange algorithm [5,18,19]. The training set design is a combinatorial optimization problem for which genetic algorithms [20-22] are particularly suitable. Genetic algorithms use a population of candidate solutions that are represented as binary strings of 0s and 1s, this population evolving toward better solutions. At each iteration of the algorithm, a fitness function is used to evaluate and select the elite individuals and subsequently the next population is formed from the elites by genetically motivated operations such as crossover and mutation. Since genetic algorithms are particularly suitable for optimization of combinatorial problems, we have used one here. It should be noted that the solutions obtained by a genetic algorithm are usually sub-optimal and different solutions can be obtained given a different starting population of candidate solutions.

In the following section, we evaluate our training population design scheme by fitting a semi-parametric mixed model (SPMM) [23,24] using the genotypes and phenotypes in the training set and calculating the correlation of the test set phenotypes to their predictions based on this model. In these mixed models, genetic information, either pedigree- or marker-based, is used to construct an additive relationship matrix. These models have been successfully used to predict breeding values in plants and animals.

A SPMM for the *n*×1 response vector ***y*** is expressed as:

$$ \boldsymbol{y}=\mathbf{X}\boldsymbol{\beta}+\mathbf{Z}\boldsymbol{g}+\boldsymbol{e}, $$

where **X** is the *n*×*p* design matrix for the fixed effects, ***β*** is a *p*×1 vector of fixed effects coefficients, **Z** is the *n*×*q* design matrix for the random effects; the random effects (***g***^′^,***e***^′^)^′^ are assumed to follow a multivariate normal distribution with mean ***0*** and covariance

$$\left(\begin{array}{cc} {\sigma^{2}_{g}} \mathbf{K} & \boldsymbol{0} \\ \boldsymbol{0} & {\sigma^{2}_{e}} \mathbf{I}_{n} \end{array} \right), $$

where **K** is a *q*×*q* relationship matrix. To fit the mixed models, we developed and used the EMMREML package [25]. The optimization algorithm was also implemented as an R package called STPGA [26]. Both are available in R [27]. All other software was also programmed in R and is available in Additional file 1.

An additive relationship matrix can be calculated from the centered scaled marker genotype matrix **M** as **K**=**MM**^′^/*m*. Given a similarity matrix **K**, the principal components used in our algorithm can be calculated from this matrix. Therefore, the statistic in Equation (4) can also be used in cases where only a similarity matrix is available.

For the examples in the next section, we fixed *λ* at 1/*m*. Although this choice is somewhat arbitrary (corresponds to a heritability value of 1/2), our method is robust to the choice of this parameter. Forty principal components were used for the approximation.

A training set of size *n*_*Train*_ in a candidate set of size *n* can be identified with a *n*−vector of 0’s and 1’s, where a 1 at a locus means that the corresponding individual in the candidate set is in the training set. Therefore, all candidate solutions to the optimization problem are vectors of length *n* with a total of *n*_*Train*_ 1’s. The genetic algorithm that we have applied starts with a random set of such solutions and generates new solutions based on one locus crossover event between two randomly selected parent solutions followed by a single random mutation event (which replaces a 1 to 0 and a 0 to 1) that occurs with probability 0.5. We used 300 iterations of the genetic algorithm with population size 800 (which amounts to evaluating 300∗800 solutions) and selection intensity 5/800 at each iteration. The training set with the best PEV measure was taken as the optimized training population at the last iteration. We decided to stop the iterations at 300 because no improvement in the criterion was observed after about 200 iterations. The solutions from the genetic algorithm may be suboptimal. To overcome this, in practice, the algorithm can be run many times, and the individuals that have been most often included can be used as the training set.

## Results

To illustrate our method, we used several datasets of different origins. The Arabidopsis dataset was published by Atwell et al. [28] and is available at cynin.gmi.oeaw.ac.at/home/resources/atpolydb. The wheat data was downloaded from www.triticeaetoolbox.org. The rice data was published in [29] and was downloaded from www.ricediversity.org/data. The maize data set was obtained from [30].

Accuracies were obtained by calculating the Pearson’s correlation coefficient (*r*) between the raw phenotypic values and the GEBV for the individuals in the test data set. The accuracies were not adjusted for trait heritabilities.

In the first two examples, the test individuals were sampled from the same population as the candidate set of individuals. Remaining examples deal with cases in which the distributions of the individuals in the test set and candidate set were not the same.

### **Example1**.

The Arabidopsis data set consisted of individuals from 199 inbred lines along with observations on 107 traits. Here, we report results for 49 of these traits. The genotype data set included 216 130 genome-wide markers.

**Figure 1:**
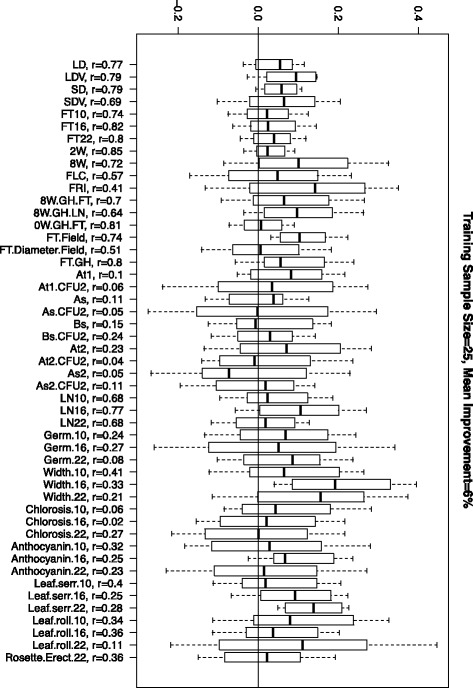
Arabidopsis data. The difference between the accuracies of the models trained on optimized populations versus random samples. Positive values indicate the cases for which the optimized population performed better as compared to a random sample.

For each trait, first a test sample of size *n*_*Test*_=50 was identified. From the remaining individuals, *n*_*Train*_=25,50 and 80 were selected in the training population by random sampling or by the optimization method described in the Methods section. The accuracies of the models were calculated by comparing the GEBV with the observed phenotypes. This was repeated 50 times and the results are summarized in Figure 1 (Also see Additional file 2: Figures S1 and S2). At all sample sizes and for almost all traits, the optimized samples improved accuracies compared to random samples. In general, the difference was larger for smaller sample sizes and it decreased as the sample size increased. The median improvements of accuracies were 6,3.4 and 2.6*%* for the sample sizes 25,50 and 80, respectively.

### **Example2**.

The rice diversity panel consisted of 400 diverse accessions of inbred lines of rice (O. Sativa) from 82 countries, including many landraces, representing all the major rice growing regions of the world. This panel was genotyped with a 44-K SNP chip. Two years (2006 and 2007) and two replicates were used to evaluate each line for important agronomic traits. This data was first presented in [29] and was also analyzed in [31]. A more detailed description of the accessions and geographical distribution of the rice germplasm is in [29]. We selected six of these traits for our analysis, namely florets per panicle (FP), panicle fertility (PF), seed length (SL), seed weight (SW), seed surface area (SSA) and straighthead susceptibility (SHS) and used the phenotypic means of each inbred line across years and replicates.

**Figure 2:**
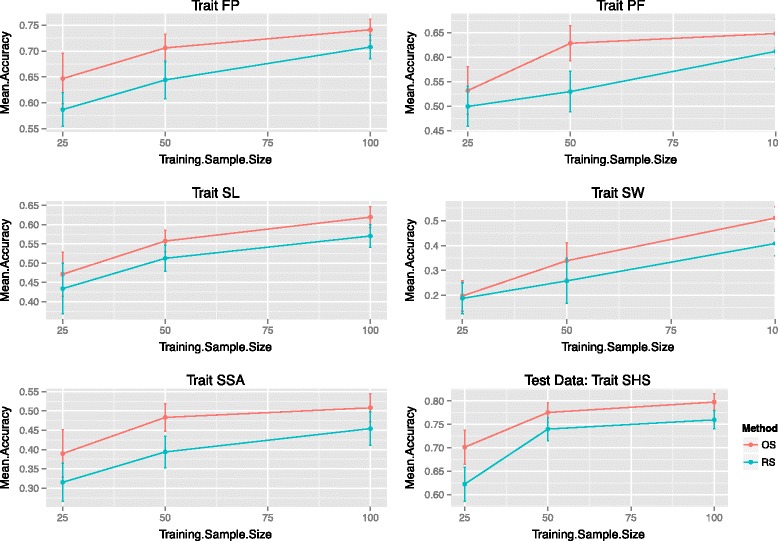
Rice data: accuracies. Comparisons of mean accuracies (measured by correlation) for the traits florets per panicle (FP), panicle fertility (PF), seed length (SL), seed weight (SW), seed surface area (SSA) and straighthead susceptibility (SHS) for different training sample sizes. Error bars at three standard error units are also included. Optimized samples outperform random samples almost exclusively.

**Figure 3:**
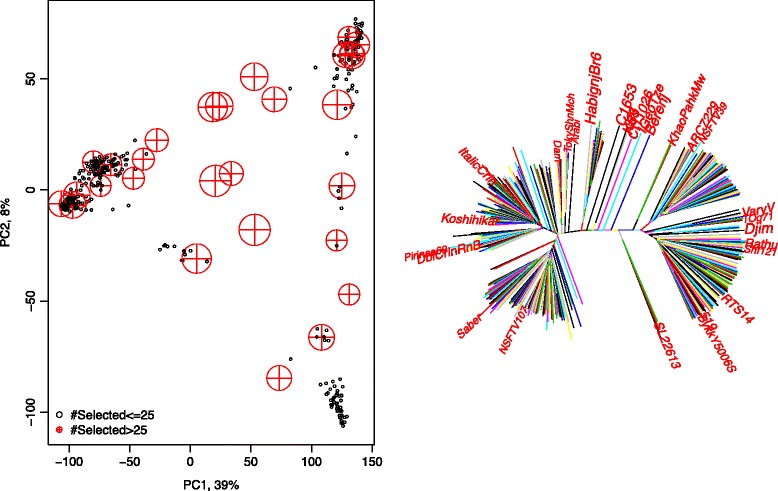
Rice data: structure. Left: Summary of the rice genotypic data with the first principal components and display lines that were most frequently selected by the optimization algorithm. Right: Lines that were most frequently selected by the optimization algorithm displayed on a neighbor-joining tree based on a genotypic distance matrix.

For each trait first, a test sample of size *n*_*Test*_=100 was randomly chosen. From the remaining individuals, *n*_*Train*_=25,50 and 100 were selected in the training population by random sampling or by the optimization method described in the Methods section. This was repeated 50 times and the results are summarized in Figures 2 and 3.

The left plot in Figure 3 represents the relationships between the individuals in the test dataset and the individuals that are most frequently selected in the training set by the first two principal components of the marker dataset. The right graph is a neighbor-joining tree based on the genotypic distance matrix, where each node represents a genotype and the distances between the nodes are indicators of the dissimilarities of the genotypes. On this neighbor-joining tree, we highlighted the lines that were most frequently selected in training sets.

As shown in Figure 3, the optimization algorithm tends to select individuals that are on average similar to individuals in the test data set (which amounts to selecting individuals that are close to the overall mean or sub-population means), but at the same time it reaches some degree of diversity in the training set.

The accuracies of the genomic selection models tended to decrease as the training and test populations diverged. In each of the examples below, accuracies were better with the optimized samples for test sets of individuals which were not random samples from the same population from which the candidate sets were selected.

### **Example3**.

**Figure 4:**
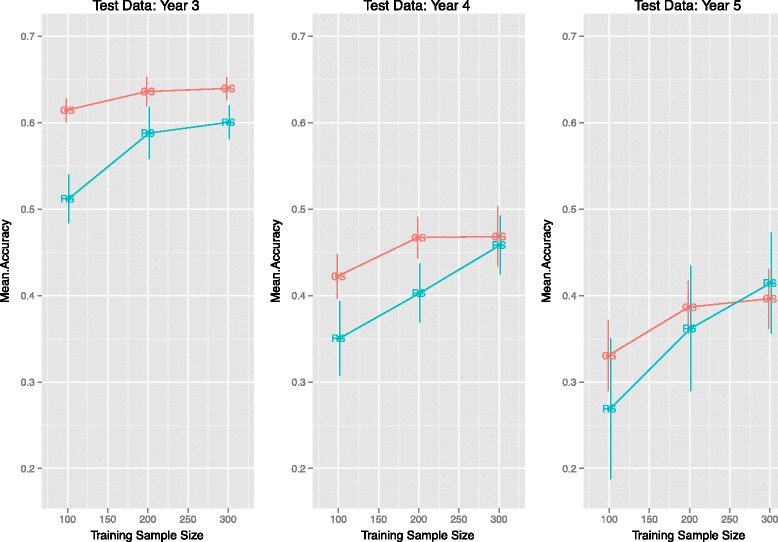
Wheat data. Comparisons of the mean accuracies (measured by correlation) when the test data set is selected from years 2007 through 2009 for different training sample sizes. For each of these cases, the training set was selected from the individuals in the years preceding the test year. Error bars at three standard error units are also included.

A total of 5087 markers for 3975 elite wheat lines in the National Small Grains Collection (NSGC) were used. In this experiment, the thousand kernel weights were observed for non-overlapping subsets of individuals over five years (108 individuals in 2005, 416 in 2006, 281 in 2007, 1358 in 2008 and 1896 in 2009). Our aim was to calculate GEBV of the individuals for each of the years 2007 to 2009 based on the individuals that were observed before that year. Genomic estimated breeding values for a random sample of *n*_*Test*_=200 individuals in the current year were estimated using first a random sample and then an optimized sample of sizes *n*_*Train*_=100 or 300 individuals and phenotypes from the years preceding the test year. The experiment was repeated 50 times and the results are summarized in Figure 4. The plots in Figure S3 (see Additional file 2: Figure S3) summarize the genotypic data with the first principal components and display the individuals that were most frequently selected by the optimization algorithm for specific test years.

The results on accuracies were similar to those obtained in the previous examples: models from optimized samples outperformed the models from random samples of the same size, but this difference decreased as the training sample size increased. The plots in Figure S3 (see Additional file 2: Figure S3) show that the selection scheme prefentially selected different individuals when the test set was varied.

### **Example4**.

In this example, we evaluated the ability of estimating GEBV across clusters in a highly structured Maize data set. This data is described in [30] and was also analyzed in [31]. The data set consists of 68 120 markers on 2 279 USA national inbred maize lines and their phenotypic means for degree days to silking. First, we divided the data into five clusters using the Euclidean distance matrix and the Ward’s criterion for hierarchical clustering. The numbers of individuals in the resulting clusters were 1317, 184, 552, 95 and 131 in the first, second, third, fourth and fifth clusters, respectively.

**Figure 5:**
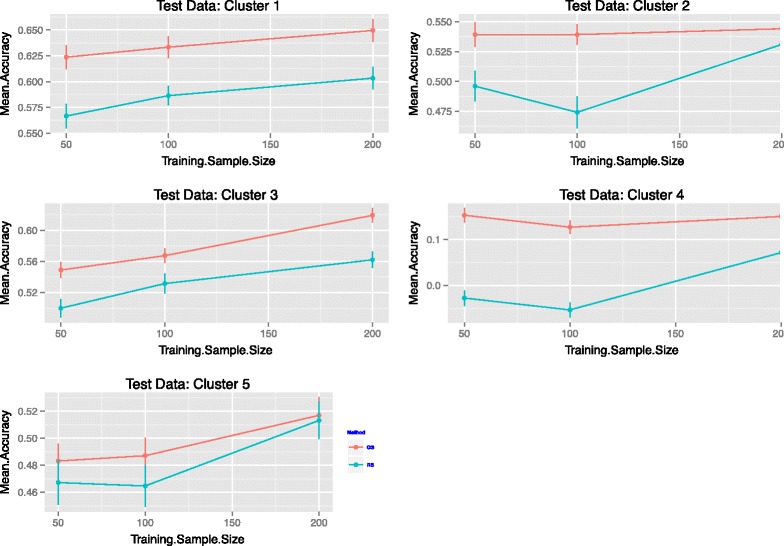
Maize data. Comparisons of the accuracies for prediction across clusters in the highly structured Maize data set. Test data set of size *n*_*Test*_=50 was selected at random in a particular cluster and a training population of size *n*_*Train*_=50,100,200 individuals was selected from the remaining clusters. Error bars at three standard error units are also included.

From each of these clusters, a test data set of size *n*_*Test*_=50 was selected at random and a training population of size *n*_*Train*_=50,100 and 200 individuals from the remaining clusters was selected by random sampling or with the optimization scheme recommended in this article. The accuracies of GEBV for the trait values in each of these clusters were calculated for 50 independent replications, and are summarized in Figure 5. Accuracies varied significantly from cluster to cluster; but, on average, the optimized training set performed better. The plots in Figure S4 (see Additional file 2: Figure S4) summarize genotyping data with the first principal components and show which lines were the most frequently selected by the optimization algorithm for each cluster as test data sets.

Although the results obtained in the examples described here suggest that accuracies can be improved by our method, not all of these improvements were statistically significant. Results should be interpreted as suggestive trends.

## Discussion

In this article, we address the training design problem and based on examples, and we show that incorporating genetic information in the test set when available can improve the accuracies of prediction models and that our method is computationally efficient.

Our results show that the accuracy of the prediction models can be improved if the individuals in the training population are selected using our scheme, especially when the required training sample size is small. Models built based on optimized samples are usually more accurate compared to models built based on random samples of larger sizes. Larger training sample sizes tend to increase accuracy, but simulations suggest that, in some cases, small training sample sizes can be just as accurate [32]. The conditions under which small training sizes retain full accuracy or possibly increase it, were not explored here since our main purpose was to select a sample size that was dictated by a given phenotyping budget. Another related scenario that was outside the context of this article involves identifying individuals that should be further phenotyped when there is already some phenotypic information about these individuals. We intend to address these questions in future studies.

In our examples, we selected the training populations separately for each trait mainly because a different subset of individuals was observed for different traits in the data sets. In practice, it would be better to select a single training population for all the traits with similar heritabilities because in real conditions phenotyping comes after this step and the procedure is robust to the choice of the shrinkage parameter *λ*, which is a function of heritability. The robustness was verified by trying different *λ* values in the algorithm and comparing the resulting training sets and it was found to be in line with the conclusions in [5].

Our method is useful when a breeding program can only phenotype a subset of the available genotyped individuals, but aims at evaluating the breeding value of a (possibly much larger) group of genotypes. Genomic selection allows to estimate the breeding value of plants or animals using genotypic and phenotypic information from a training population. By replacing random sampling with our optimized selection scheme while selecting the training set, the breeding values in the test set can be estimated with higher accuracies. If the candidate and the test sets are both randomly selected from the same population, selecting an optimized training sample from the candidates with our method improves the accuracies of GEBV for this population. However, the use of our method is also limited since it requires that all the genotypes are known in advance and that the individuals that are selected in the training set are available for phenotyping.

We have discussed the training population design problem in the context of the regression of continuous traits on the genotypes based on SPMM. However, our proposed approach can be used to obtain more accurate prediction models in the general statistical learning domain. The bibliography on optimal experiment design in statistical learning is extensive [19,33,34] and review articles [18,35] provide a good survey of the area. The methods described here can be used to find optimal experiment designs for high-dimensional prediction problems where cost per individual of measuring or analyzing the response variable is too high, and therefore, a small number of training examples is required. They are also particularly useful when the candidate set from which the training set must be chosen is not representative of the test data set.

Our results indicate that the genetic algorithm scheme adopted here is very efficient at finding a good solution in the training population design problem. However, there is no guarantee that the solutions found by this algorithm are globally optimal solutions. Since the purpose of the article was to evaluate the overall improvement over many replications of the same experiments, we could not afford to start the genetic algorithm at several different starting points but it would be safer to do so. After several runs of the algorithm, we recommend picking the solution that led to the best value of the utility function or the individuals that were most frequently selected in the training set for the final training set.

A dynamic model building approach might be more suitable when the individuals in the test set are structured. An optimized set of individuals has a better chance of representing the sub-populations compared to a random sample of the same size. Accuracies can be improved by using different models for different parts of the test set, built on the basis of a subset of individuals that are chosen from the candidate set by the training population design algorithm. We intend to explore this and some related issues in a follow-up article.

## Additional files

Additional file 1
**R Code.** R programs for replicating the analysis in the paper.

Additional file 2
**Figure S1.** Arabidopsis data, sample size = 50. Differences between accuracies from optimized model versus random samples for sample size = 50. **Figure S2.** Arabidopsis data, sample size = 80. Differences between accuracies from optimized model versus random samples for sample size = 80. **Figure S3.** Wheat datacore. Genotypes selected most frequently by the optimization algorithm. **Figure S4.** Maize data core. Genotypes selected most frequently by the optimization algorithm.
