# Supplementary material for: Optimization of genomic selection training populations with a genetic algorithm
Source: Genet Sel Evol. 2015 May 6;47(1):38. doi: 10.1186/s12711-015-0116-6 (PMC4422310; doi:10.1186/s12711-015-0116-6)
Supplement: Additional file 2 — Figure S1. Arabidopsis data, sample size = 50. Differences between accuracies from optimized model versus random samples for sample size = 50. Figure S2. Arabidopsis data, sample size = 80. Differences between accuracies from optimized model versus random samples for sample size = 80. Figure S3. Wheat datacore. Genotypes selected most frequently by the optimization algorithm. Figure S4. Maize data core. Genotypes selected most frequently by the optimization algorithm. [file 12711_2015_116_MOESM2_ESM.pdf]

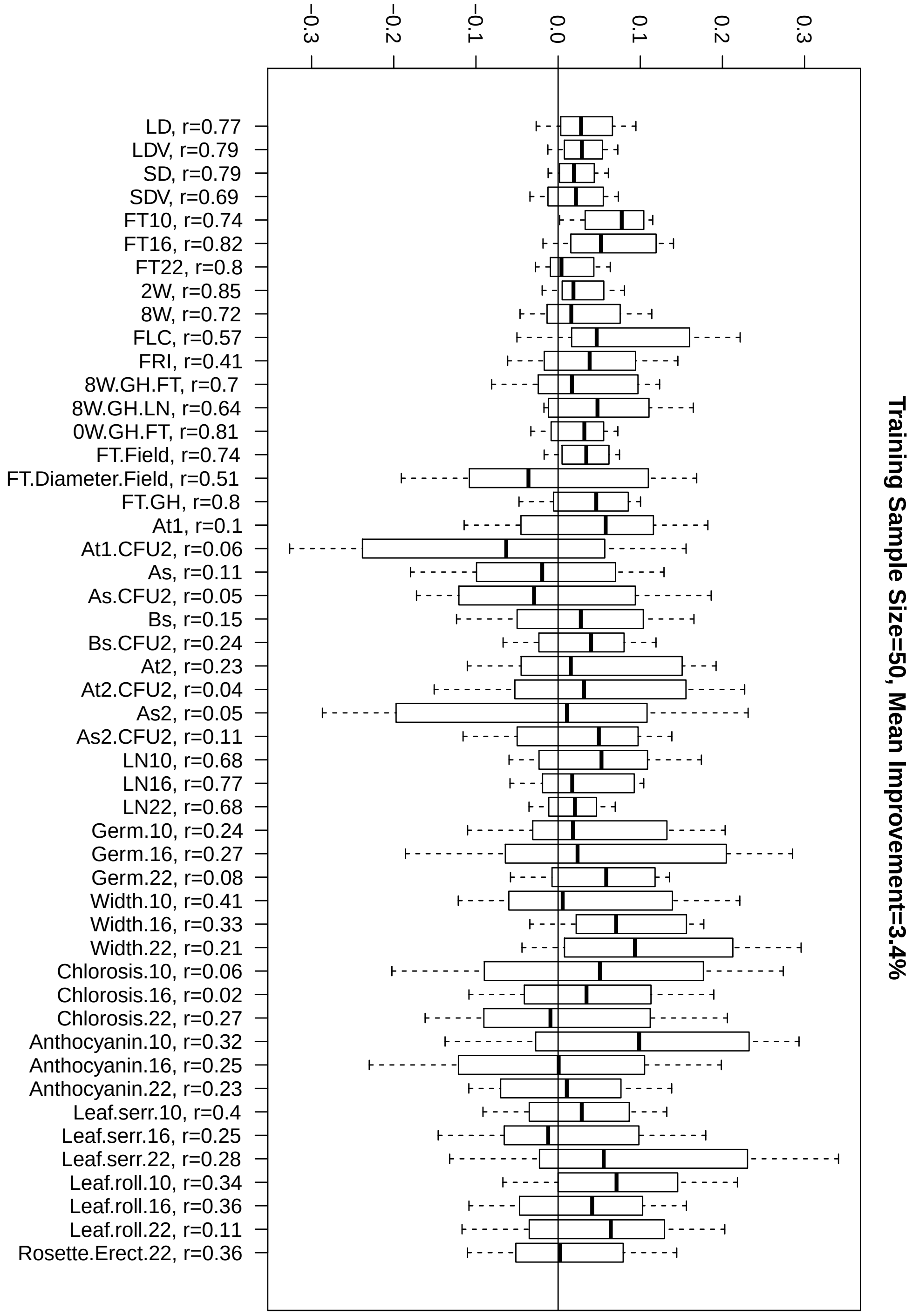

Figure S1: Arabidopsis data, sample size = 50.

Differences between accuracies from optimized model versus random samples for sample size =50 in Arabidopsis. Positive values indicate better performance of the optimized model compared with random sample.

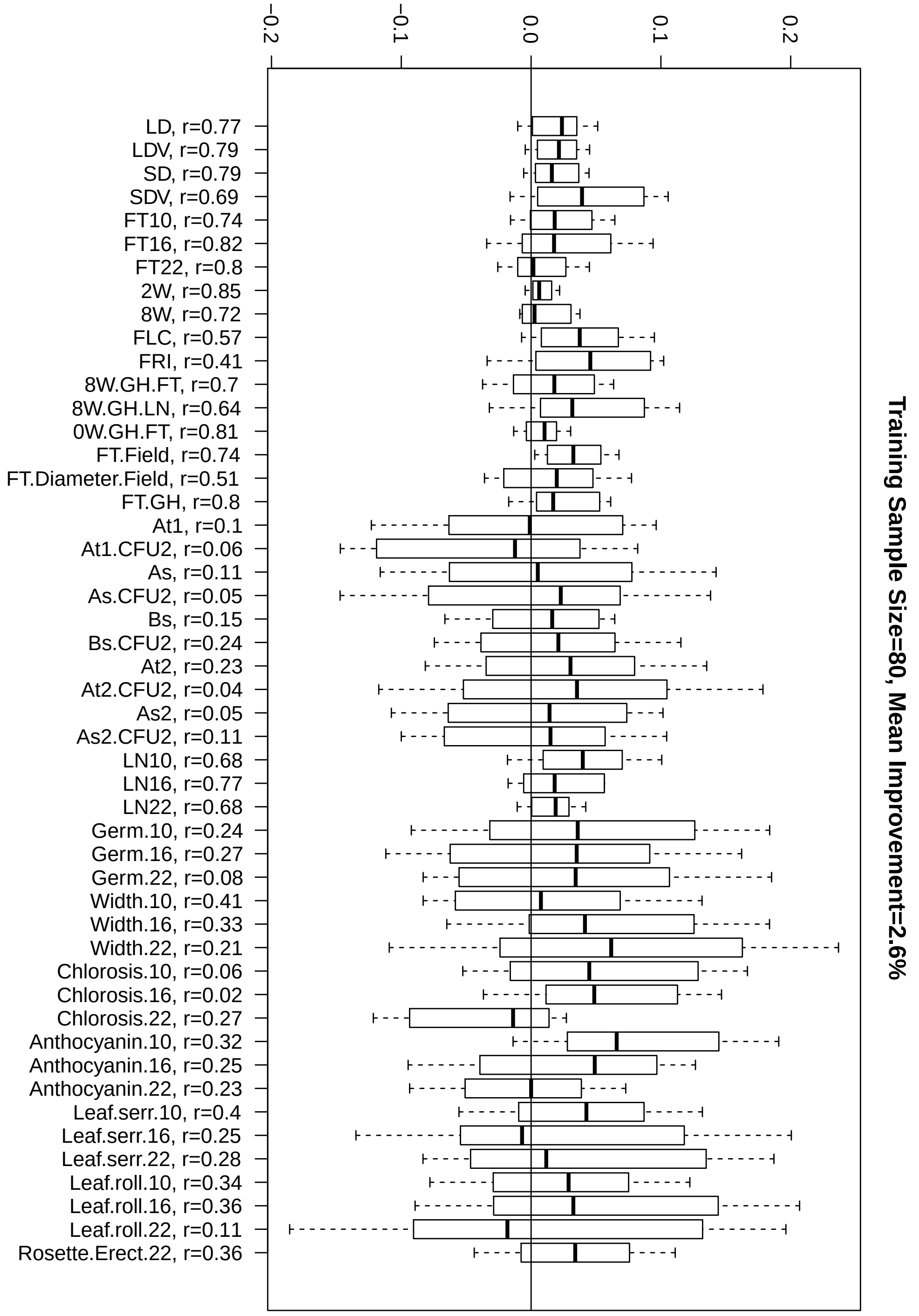

Figure S2: Arabidopsis data, sample size = 80.

Differences between accuracies from optimized model versus random samples for sample size =80 in Arabidopsis. Positive values indicate better performance of the optimized model compared with random sample.

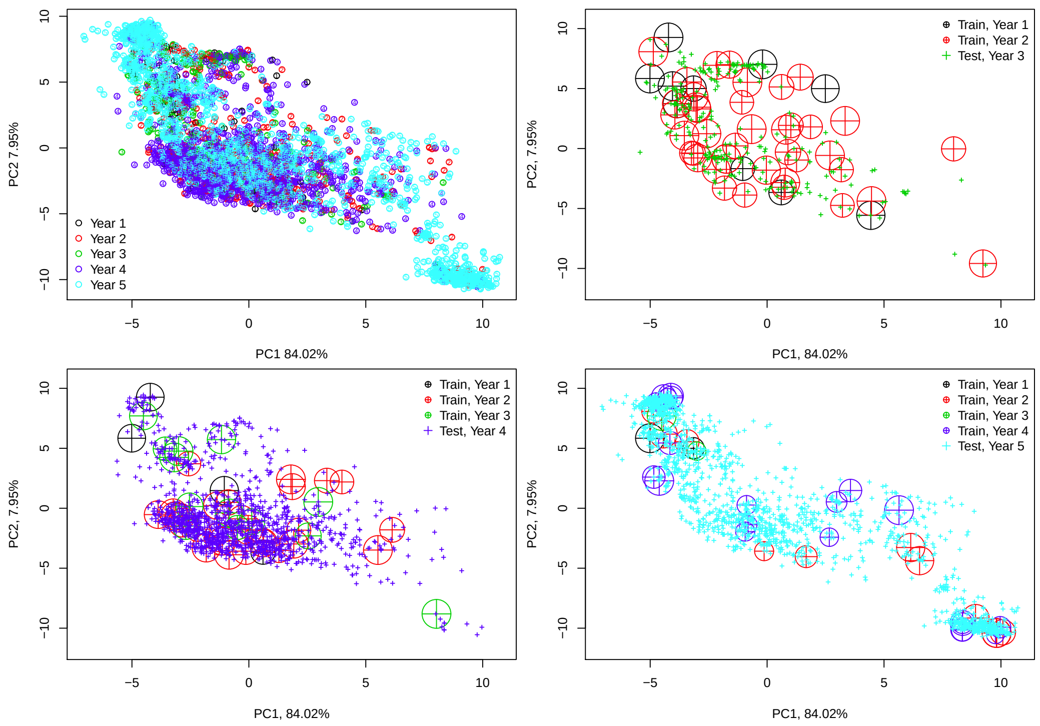

Figure S3: Wheat data core.

Genotypes selected from the optimization algorithm over the fifty run are plotted on the first two principal components analysis in the wheat dataset. Top left: All 5 years are distinguished by colors. Top-right: the training set is built from Year 1 and 2. Predictions are made in year 3. Bottom-right: the training set is built from Year 1,2 and 3. Predictions are made in year 4. Bottom-left: The training set is built from Year 2, 3 and 4. Predictions are made in year 5. Individuals that were not selected more than 25 times are excluded. The size of the circle is proportional to the frequency of selection of that individual from the algorithm. .

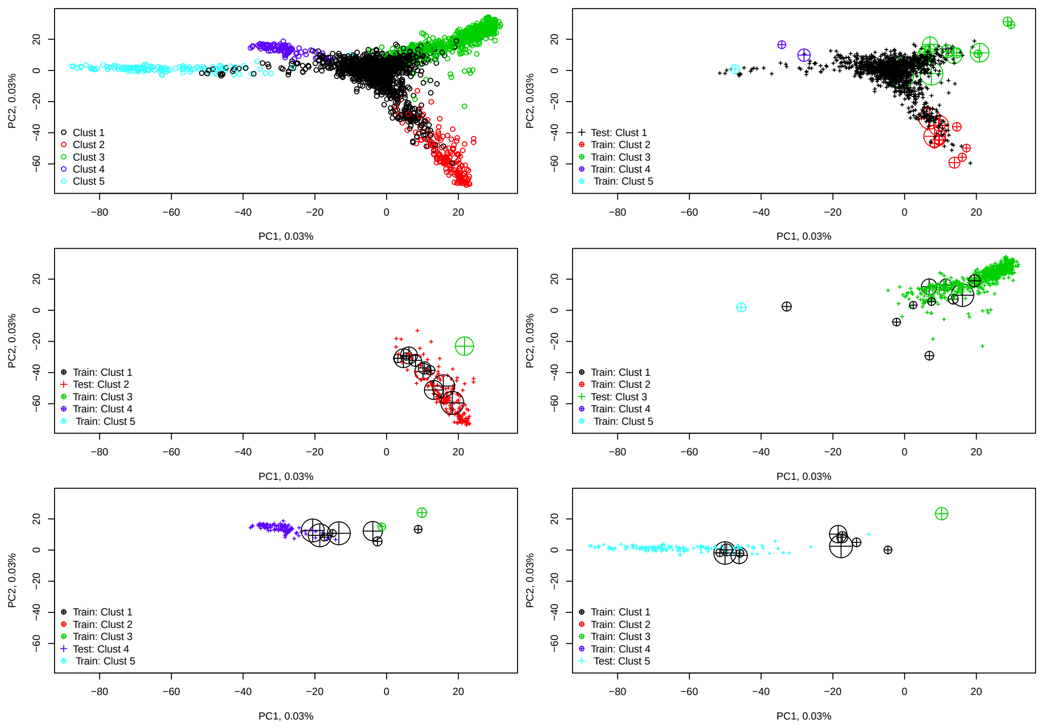

Figure S4: Maize data core.

Genotypes selected from the optimization algorithm over the fifty run are plotted on the first two principal components analysis in the maize dataset. Top left: All 5 clusters are distinguished by colors. Top-right: Predictions are made in Cluster 1, the remaining clusters set up the training set. Middle-right: Predictions are made in Cluster 2, the remaining clusters are the training set. Middle-left: Predictions are made in Cluster 3, the remaining clusters are the training set. Bottom-right: Predictions are made in Cluster 4, the remaining clusters are the training set. Bottom-left: Predictions are made in Cluster 5, the remaining clusters are the training set. Individuals who were not selected more than 25 times are excluded. The size of the circle is proportional to the frequency of selection of that individual from the algorithm.
